# Incomplete Uterine Rupture During the Second Trimester of Pregnancy, Successful Management With Continued Gestation Until 37 Weeks: A Case Report

**DOI:** 10.7759/cureus.77885

**Published:** 2025-01-23

**Authors:** Alexandros Psarris, Antonia Varthaliti, Anthi-Maria Papahliou, Marianna Theodora, Charis Bourgioti, Vasilios Lygizos, Maria Anastasia Daskalaki, Panagiotis Antsaklis, Vasileios Agorogiannis, Andreas Pampanos, Pelopidas Koutroumanis, George Daskalakis

**Affiliations:** 1 1st Department of Obstetrics and Gynecology, Alexandra Maternity Hospital, National and Kapodistrian University of Athens, Athens, GRC; 2 Department of Radiology, Aretaieion University Hospital, National and Kapodistrian University of Athens, Athens, GRC; 3 Obstetrics and Gynecology, National and Kapodistrian University of Athens, Athens, GRC; 4 Department of Surgery, University Hospital of Larissa, Larissa, GRC; 5 Department of Genetics, School of Medicine, National and Kapodistrian University of Athens, Athens, GRC; 6 1st Department of Obstetrics and Gynecology, School of Medicine, National and Kapodistrian University of Athens, Athens, GRC

**Keywords:** obstetrics, perinatal outcome, pregnancy, second trimester, uterine rupture

## Abstract

Uterine rupture is rare but one of the most severe and fatal complications in obstetrics. Nonetheless, its presence in the second trimester is exceptionally rare, posing considerable difficulties for diagnosis and management. This case report demonstrates a 38-year-old woman with a history of a previous cesarean delivery who presented at 18 weeks of gestation at the emergency department with severe, acute onset abdominal pain at the right iliac fossa. The patient underwent surgery due to high suspicion, and incomplete uterine rupture was revealed and managed appropriately. Following the surgery, the patient was admitted to the hospital for close monitoring, and at 37 weeks, a successful cesarean delivery resulted in the birth of a live male infant. This case highlights that the obstetric team should remain alert to recognize early the signs and symptoms of this complication in patients with a history of uterine surgery and, as a result, to achieve an optimal perinatal outcome. Specific management and monitoring algorithms could aid the obstetric team in optimally treating these patients.

## Introduction

Uterine rupture is an uncommon but critical obstetric emergency, presenting considerable dangers to both the mother and the fetus. It manifests in fewer than 0.05% of pregnancies and is more prevalent in the third trimester or during labor as a result of heightened uterine pressure and contractions [1]. Uterine rupture in the second trimester is exceedingly uncommon, posing distinct challenges in the diagnosis and treatment [2].

Uterine rupture is associated with previous uterine procedures, like cesarean sections or myomectomy, which undermine the structural integrity of the uterine wall [3]. Supplementary risk factors encompass uterine abnormalities, multiparity, and connective tissue disorders [4]. In resource-limited environments, adolescent pregnancy, low socio-economic position, and unregulated labor significantly elevate the risk of rupture [5]. This condition usually manifests with abrupt and severe abdominal pain, hemoperitoneum, and fetal distress in the third trimester; however, second-trimester instances may exhibit unclear or absent symptoms, complicating prompt diagnosis [6]. Uterine rupture in the second trimester presents even more diagnostic and management challenges compared to the third-trimester cases, as there is a lack of evidence in the literature.

Ultrasound and magnetic resonance imaging (MRI) are indispensable for diagnosing uterine rupture; however, they are not always conclusive. A timely diagnosis and care necessitate a heightened index of suspicion, which is influenced by the patient's clinical history and symptoms [7]. Prompt surgical intervention is crucial for protecting maternal and fetal well-being and managing hemorrhage [8,9].

This case report aims to illustrate a case of incomplete uterine rupture in the second trimester of a 38-year-old woman with a history of previous cesarean section and to propose a way of effective management. Despite the severity of the disease, the pregnancy was successfully maintained through prompt surgery and vigilant monitoring, which led to the delivery of a healthy full-term neonate at thirty-seven weeks of gestation. This report emphasizes the importance of interdisciplinary care in addressing this uncommon complication and provides valuable insights into the current literature.

## Case presentation

**Figure 1:**
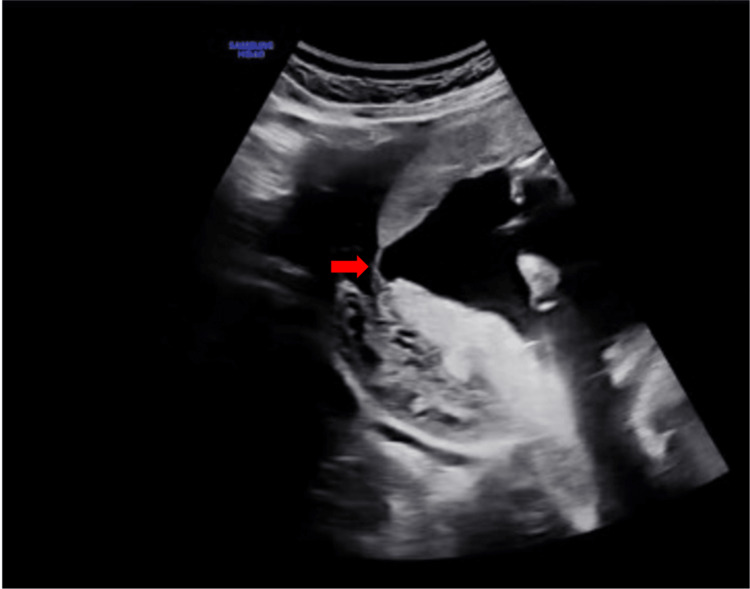
Ultrasound image showing the incomplete uterine rupture (red arrow showing that the myometrium is disrupted)

A 38-year-old lady, gravida II, para I, arrived at the emergency department of an outside hospital at 18 weeks of gestation with acute onset right iliac fossa pain and signs of acute abdomen. Her obstetric history comprised one prior cesarean section three years ago, subsequent to curettage for significant puerperal hemorrhage. The patient's medical history includes rheumatoid arthritis in remission, for which she is in long-term treatment with methylprednisolone and azathioprine, and the patient has denied any recent trauma or vigorous activity. The patient was hemodynamically stable. The abdominal examination indicated pain in the right iliac fossa, confirming a positive McBurney's sign, along with signs of peritonism. A transabdominal ultrasound revealed an 18-week intrauterine pregnancy with a detectable heartbeat, disruption of the myometrium (Figure 1), and the presence of free fluid in the pouch of Douglas. The patient was admitted for meticulous observation and care. Laboratory testing demonstrated a reduction in hemoglobin levels (Table 1), while an MRI of the abdomen and pelvis identified free fluid measuring 6.5x4.2x3.8 cm (blood) in the peritoneal cavity, along with peritoneal fat contamination at the ileocecal junction.

**Table 1:** Blood results preoperatively Reference range: HCT: 36-48%, Hgb=12.1-15.1 g/dl HCT: Hematocrit; Hgb: Hemoglobin

Time of blood collection	HCT (%)	Hgb (g/dl)
Day 0 - 15:30 pm	33.6	11
Day 0 - 23:00 pm	27.7	9.3
Day 1 - 04:30 am	25.4	8.6

In light of the clinical suspicion, an urgent exploratory laparotomy was conducted, revealing hemoperitoneum extending from the right iliac fossa to the subhepatic area, along with an incomplete uterine rupture of 3 cm at the right side of the anterior uterine wall close to the fundus, 1 cm above the right fallopian tube. At the point of the rupture, there was an adhesion with the omentum that, due to the uterine growth of the pregnancy, revealed the myometrium and caused bleeding. This was followed by symphysiolysis, hemostatic management of the incomplete uterine rupture utilizing a hemostatic agent (suturing of the uterus was not possible due to the uterine enlargement of the pregnancy), appendectomy, intestinal resection of Meckel's diverticulum, and side-to-side anastomosis employing a linear stapler. Following the procedure, a transabdominal ultrasound was conducted, revealing a positive fetal heartbeat. The patient's health stabilized in the ensuing days, and her abdominal pain diminished. She was discharged from the hospital seven days later. 

**Figure 2:**
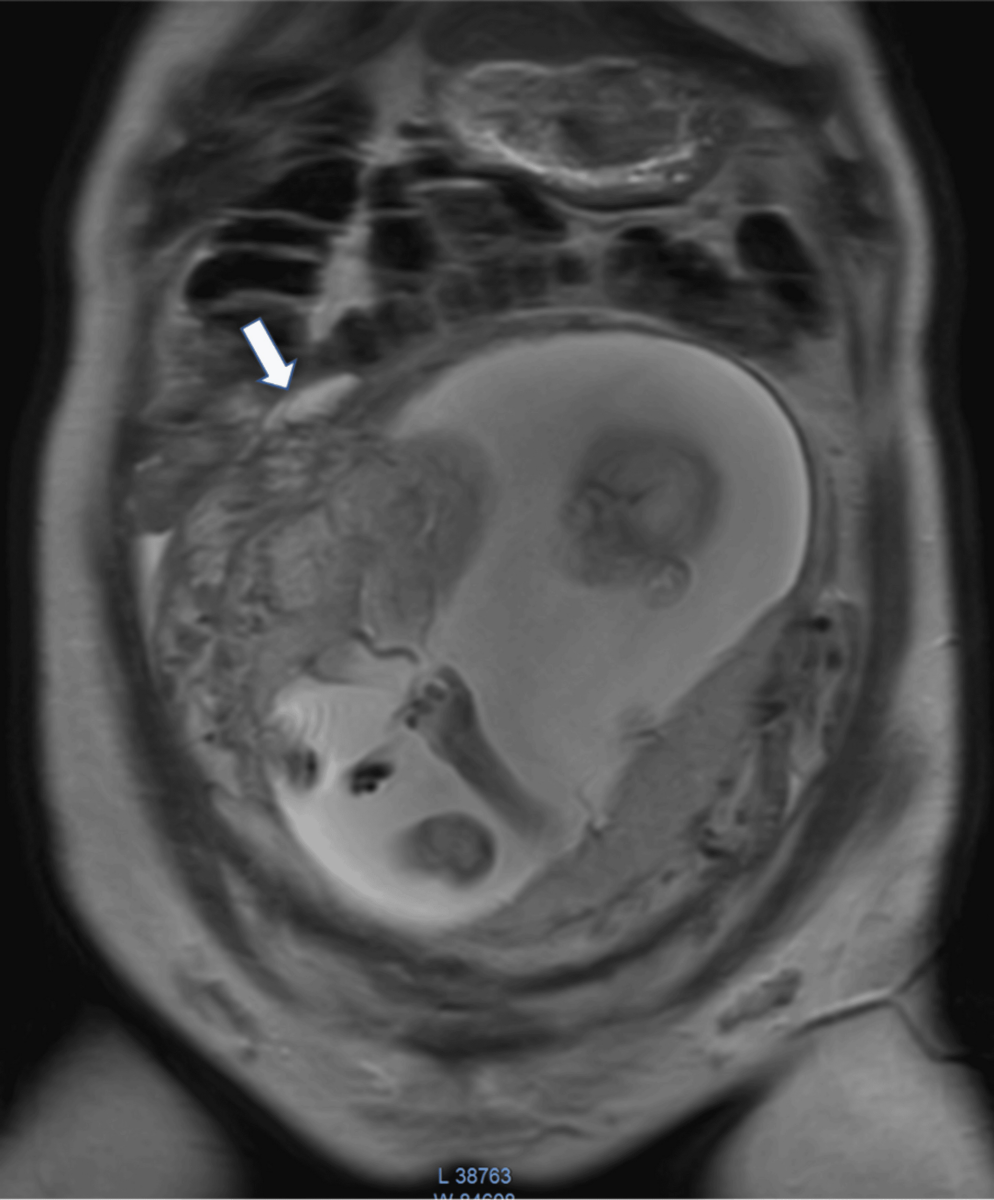
Coronal T2 weighted image shows extensive focal myometrial thinning and a small fluid collection (arrow) on the right side of the uterine fundus.

At 22 weeks of the pregnancy, the patient underwent a repeat MRI of the abdomen and pelvis, which revealed a small collection of 3.9x1.3 cm surrounding the uterus at the site of the uterine rupture (Figure 2). Anomaly scan was also performed without revealing any fetal abnormality. The patient was admitted to our hospital at 24 weeks of pregnancy for close monitoring with clinical evaluation, a non-stress test twice daily, and regular ultrasound scans two times per week. The scans showed no significant increase in free fluid, and the fetal heart rate remained within normal limits. 

**Figure 3:**
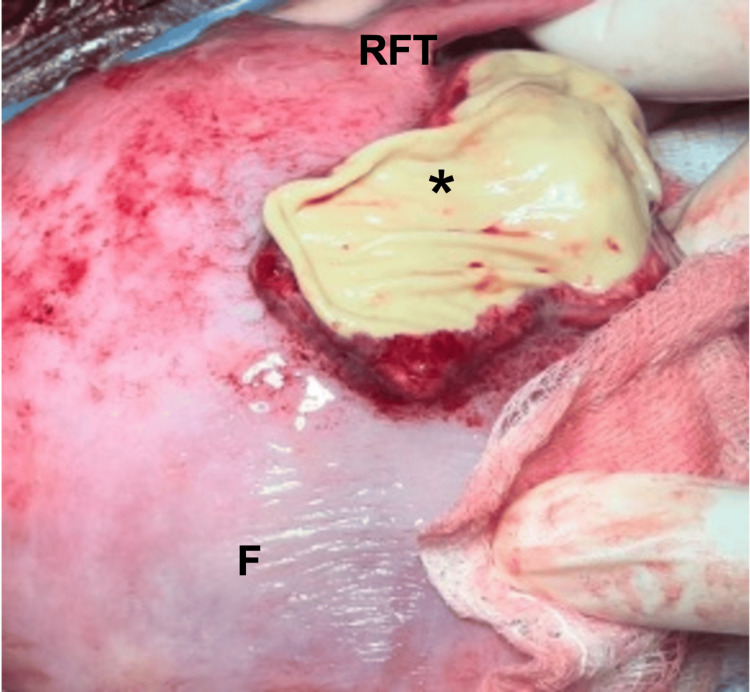
Intraoperative image during the c-section, showing the point of the incomplete uterine rupture. Asterisk: scar tissue formed at the point of uterine rupture. F: Uterine Fundus; RFT: Right Fallopian Tube

At 37 weeks of gestation, an elective cesarean section was performed to decrease the risk of uterine rupture during labor, given her history of previous cesarean delivery and the prior incident of incomplete rupture. During the cesarean section, extensive scar tissue and severe thinning of the uterine wall were seen in the fundus and anterior wall of the uterus (Figure 3).

The procedure was uneventful otherwise, and the patient delivered through the cephalic presentation: one alive male newborn 3080gr with APGAR scores of 8 and 9 at 1 and 5 minutes, respectively. Postoperatively, the patient was stable, and both mother and infant were discharged after four days. At four months, both mother and baby are alive and well.

## Discussion

Uterine rupture is a rare and fatal condition during pregnancy for the mother and the fetus. Its incidence at any gestational age is 0.05% and cannot be predicted [3]. However, sudden uterine rupture in the second trimester has been rarely documented [4].

There are various reasons that uterine rupture can occur, and they usually differ between Western and developing countries [5-6]. In Western countries, the trial of labor after cesarean section and the use of uterotonics are common causes of uterine rupture, while in developing countries, common causes of uterine rupture include multiparity, pregnancy at a younger age, poor socio-economic status, and unsupervised labor [4,7-9]. All studies agree that the main risk factor for uterine rupture, globally, is previous cesarean section. In addition, another risk factor that needs to be considered is previous uterine surgery, such as myomectomy [10]. It has also been shown that multiple pregnancies, obstructed labor, and uterine anomaly may provoke uterine rupture, even in women without a prior history of uterine surgery [11-15].

The early diagnosis of uterine rupture is of utmost importance for reducing maternal and fetal morbidity and mortality, as well as having successful pregnancy management. Typical symptoms include abdominal pain, usually of sudden onset and acute in nature, vaginal bleeding, vomiting, shock, no uterine contractions, hemoperitoneum, and fetal deterioration [16-18]. Pregnant women do not display consistent symptoms, especially in early gestation, when uterine rupture may occur asymptomatically. Moreover, symptoms may be lacking, and ultrasonography patterns are not consistently identifiable to facilitate diagnosis. Therefore, physicians should sustain an elevated clinical suspicion, guided by the patient's history and clinical symptoms. Following diagnosis, immediate surgical intervention is generally the most appropriate management [19-20]. The review from Surico et al. supports our case and illustrates that prompt management can enable delayed delivery to improve perinatal outcomes [19].

In the present case report, the decision to delay birth in this pregnant woman was based on multiple factors. First, at exploratory surgery, there was an incomplete uterine rupture, which was properly managed. Second, the fetus was not in distress, with a detachable heartbeat and no evidence of impending birth. Third, the pregnant woman was closely monitored until week 37, with all tests showing stable pregnancy. It is understood that the patient could go into labor at any time, rendering the monitoring of the patient quite difficult. In addition, if the patient went into labor or if the uterus ruptured unexpectedly, this could have resulted in a devastating situation. Nonetheless, the decision to delay pregnancy was based on all available data and considered the best at that time.

## Conclusions

This case report shows that incomplete uterine rupture can occur during the second trimester of pregnancy, even though it's rare. Although it remains uncertain whether it should be managed conservatively or with cesarean section, repair, or hysterectomy, this case report highlights the feasibility of delaying delivery to term after a uterine rupture in the second trimester, with close monitoring and collaboration of a multidisciplinary team. It is crucial that the obstetric team should maintain a high degree of suspicion for this complication in patients with a history of uterine surgery and be able to recognize the signs and symptoms early. Dedicated management and monitoring algorithms should be implemented to treat these patients. In addition, hospitals need to establish focused, highly-trained teams that can identify these patients and treat them in a timely manner. Future research is needed to develop evidence-based guidelines for managing pregnancy complications related to uterine rupture to improve maternal and fetal outcomes.
